# Supplementary material for: Activity‐based anorexia enhances glutamatergic synaptic transmission and neuronal excitability within the nucleus accumbens of female mice
Source: Physiol Rep. 2026 May 29;14(11):e70936. doi: 10.14814/phy2.70936 (PMC13239154; doi:10.14814/phy2.70936)

Supplemental Figures

**Supplemental Figure 1. Full Western blot for blot panels showed in Figure 2A and 2B.** GluA1 and GluA2 membrane protein levels in sedentary (S), food restricted (F), exercise (E) and ABA (A) groups.


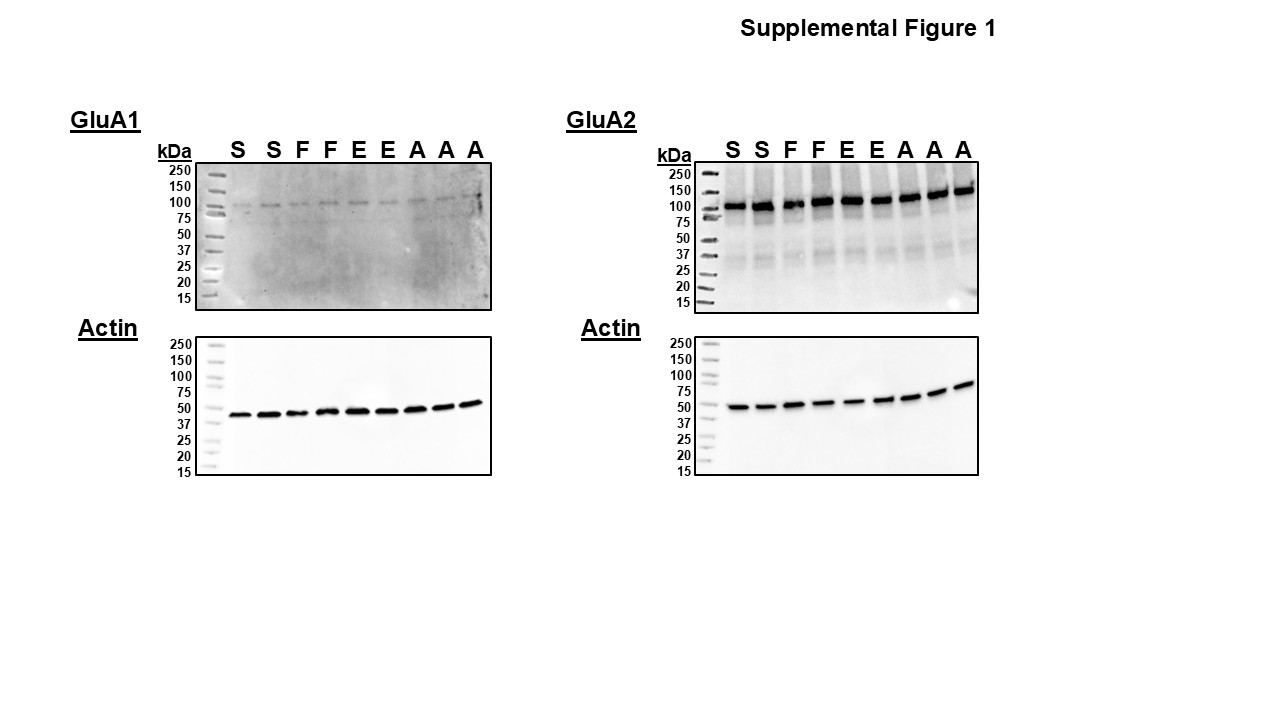

Supplement: Supplementary file 1 — Figure S1. Full Western blot for blot panels showed in Figure 2a,b. GluA1 and GluA2 membrane protein levels in sedentary (S), food restricted (F), exercise (E) and ABA (A) groups. [file PHY2-14-e70936-s001.docx]
